# The nine ADAMs family members serve as potential biomarkers for immune infiltration in pancreatic adenocarcinoma

**DOI:** 10.7717/peerj.9736

**Published:** 2020-09-30

**Authors:** Bing Qi, Han Liu, Ying Dong, Xueying Shi, Qi Zhou, Fen Zeng, Nabuqi Bao, Qian Li, Yuan Yuan, Lei Yao, Shilin Xia

**Affiliations:** 1 Department of General Surgery, The First Affiliated Hospital of Dalian Medical University, Dalian, China; 2 College of Stomatology, Dalian Medical University, Dalian, China; 3 Gastrointestinal Department, The Second Affiliated Hospital of Dalian Medical University, Dalian, China; 4 Institute (College) of Integrative Medicine, Dalian Medical University, Dalian, China; 5 Clinical Nutrition Department, Shandong Provincial Hospital Affiliated to Shandong First Medical University, Jinan, China; 6 Department of General Surgery, The Second Affiliated Hospital of Harbin Medical University, Harbin, China; 7 Clinical Laboratory of Integrative Medicine, The First Affiliated Hospital of Dalian Medical University, Dalian, China

**Keywords:** Pancreatic adenocarcinoma, ADAM, Immune infiltration

## Abstract

**Background:**

The functional significance of ADAMs family members in the immune infiltration of pancreatic adenocarcinoma (PAAD) awaits elucidation.

**Methods:**

ADAMs family members with significant expression were identified among differentially expressed genes of PAAD based on The Cancer Genome Atlas (TCGA) database followed by a verification based on the Oncomine database. The correlation of ADAMs in PAAD was estimated with the Spearman’s rho value. The pathway enrichment of ADAMs was performed by STRING and GSEALite, respectively. The protein–protein interaction and Gene Ontology analyses of ADAMs and their similar genes were exanimated in STRING and visualized by Cytoscape. Subsequently, the Box-Whisker plot was used to show a correlation between ADAMs and different tumor grade 1/2/3/4 with Student’s *t*-test. TIMER was applied to estimate a correlation of ADAMs expressions with immune infiltrates and immune checkpoint blockade (ICB) immunotherapy-related molecules. Furthermore, the effect of copy number variation (CNV) of ADAMs genes was assessed on the immune infiltration levels.

**Result:**

ADAM8/9/10/12/15/19/28/TS2/TS12 were over-expressed in PAAD. Most of the nine ADAMs had a significant correlation. ADAM8/12/15/19 expression was remarkably increased in the comparison between grade 1 and grade 2/3 of PAAD. ADAM8/9/10/12/19/28/TS2/TS12 had a positive correlation with almost five immune infiltrates. ADAM12/19/TS2/TS12 dramatically related with ICB immunotherapy-related molecules. CNV of ADAMs genes potentially influenced the immune infiltration levels.

**Conclusion:**

Knowledge of the expression level of the ADAMs family could provide a reasonable strategy for improved immunotherapies to PAAD.

## Introduction

Pancreatic adenocarcinoma (PAAD) represents 85% all pancreatic cancer and is frequently described one of the most lethal malignancies with a poor outcome, although a limited number of PAAD patients can undergo routine adjuvant therapies after surgery (Higuera et al., 2016; Neoptolemos, 2011). In recent decades, more in-depth studies empower the immunotherapy on the clinical treatment for patient with PAAD. The immunotherapy has been used for the PAAD treatment accounting for an immune regulatory in the tumor microenvironment (TME) (Fan et al., 2020). In the TME, immunocytes resemble the features of sampling the microenvironment and recognizing the antigens, which originate from tumor cell (Nazemi & Rainero, 2020). The fact that immunotherapy is successful in treatment for malignancies like PAAD pleas for a better understanding of the molecular mechanism by which tumor-associated antigens (TAAs) can be recognized and captured by immunocytes.

TAAs, often originated from cell surface, are predominantly produced by the processing of membrane-associated protease (Vigneron, 2015). It has previously been observed that cancer cells often express an elevated level of the membrane-associated metalloprotease, such as a-disintegrin-and-metalloproteinases (ADAMs) (Murphy, 2008). ADAMs is a family of transmembrane proteases, which release substrates by cleaving the ectodomains from cell surface proteins (Klein & Bischoff, 2011). ADAMs family members cover a wide spectrum of potential substrates, and various cleaved ectodomains are soluble and biologically active (Moss et al., 2016; Reiss & Bhakdi, 2017). The functional complexity of substrates enables ADAMs family as an important participant in extracellular interaction. Overexpression of ADAMs is not oncogenic, which was exemplified as ADAM9 harboring angiogenetic function without an impact on tumor cell proliferation (Oria et al., 2019), but due to a cancer characteristic of cleavage products (Herrlich & Herrlich, 2017). Some of the ADAMs family members have been reported as an important role in the progression of pancreatic cancer. For example, ADAM8 and ADAM28 were regarded as a potential therapeutic target in pancreatic cancer (Schlomann et al., 2015; Valkovskaya et al., 2007; Wei et al., 2019). ADAM10 is remarkably involved in the invasiveness and migration of pancreatic cancer cells (Sepult et al., 2019). In addition, ADAMs can serve as a specific resource to regulate the cell–cell or cell-environment interaction, such as immune infiltration. Therefore, an analysis of the ADAMs family members may offer new insights on regulation of immune infiltration in PAAD, which may optimize immunotherapeutic strategies for PAAD patient.

In the present study, we carried out an initial identification of nine ADAMs from differently expressed genes (DEGs) of PAAD based on both TCGA and Oncomine databases, followed by a correlation of analysis. The biological function and protein–protein interaction (PPI) of all nine ADAMs and their similar genes was performed. After correlation analysis of tumor grade, we studied a correlation of ADAMs expression with immune infiltration in PAAD at molecular and immunocyte levels. Furthermore, we continued to examine the copy number variation (CNV) of ADAMs to explore the potential factors affecting the association between ADAMs and immune infiltration. This integrated analysis has facilitated biological insights of ADAMs family members in PAAD, contributing to influence PAAD clinical management, especially the immunotherapeutic strategies.

## Materials & Methods

### Identification of differently expressed genes in PAAD

GEPIA (http://gepia.cancer-pku.cn/index.html) is an interactive web tool for analyzing the RNA sequencing expression data of 9,736 tumors and 8,587 normal samples from the TCGA. It can provide a customizable function to analyze the expression profiling of genes between tumor and normal samples (Tang et al., 2017). In this study, GEPIA was used to analyze the expression of nine ADAMs family members in PAAD. Differentially expressed ADAMs were identified with higher —log_2_ Fold change (FC)— and lower q values than a pre-set threshold (—log_2_ FC—> 2 and *q* < 0.01).

Oncomine database (https://www.oncomine.org) is a web resource to provide the different expression profile of genes from gene-wide expression analysis (Rhodes et al., 2004). In our study, we performed a summary view in PAAD to verify the differently expressed nine ADAMs family members from TCGA database. We set a threshold including *P*-value as 0.01, Fold change as 1.5, and gene rank as 10%.

Data are available at TCGA https://portal.gdc.cancer.gov/projects/TCGA-PAAD (search terms: TCGA-CESC and TCGA-PAAD) and Oncomine (target genes isolated and collected using TCGA and Oncomine are available in Tables 1 and 2, separately).

**Table 1:** Different expression of nine ADAMs family members in PAAD patients.

**Gene symbol**	**Log**_**2**_**FC**	***q*-value**
ADAM9	3.53	2.06E−70
ADAM28	3.433	1.06E−52
ADAM8	3.111	2.67E−54
ADAMTS2	2.94	4.20E−60
ADAM19	2.925	1.04E−62
ADAMTS12	2.674	8.86E−57
ADAM12	2.538	1.02E−46
ADAM10	2.475	3.42E−57
ADAM15	2.194	1.07E−48

**Table 2:** Significant changes of ADAMs expression in transcription level between PAAD and normal pancreas tissues.

	**Types of PAAD vs. Pancreas**	**Fold change**	***P* value**	***t*-test**	**Ref**
ADAM9	Pancreatic Adenocarcinoma	4.592	1.76E−4	5.704	Logsdon Pancreas [27]
	Pancreatic Adenocarcinoma	3.656	1.84E−12	8.572	Badea Pancreas [78]
	Pancreatic Adenocarcinoma	2.621	5.16E−4	5.084	Lacobuzio-Donahue Pancreas 2 [36]
ADAM28	Pancreatic Adenocarcinoma	3.772	2.17E−8	6.403	Badea Pancreas [78]
ADAM8	Pancreatic Adenocarcinoma	3.484	1.50E−7	9.589	Logsdon Pancreas [27]
	Pancreatic Adenocarcinoma	5.108	0.002	3.256	Grutzmann Pancreas [25]
	Pancreatic Adenocarcinoma	3.756	5.58E−5	5.298	Lacobuzio-Donahue Pancreas 2 [36]
ADAMTS2	Pancreatic Adenocarcinoma	1.805	5.01E−5	5.761	Buchholz Pancreas [38]
	Pancreatic Adenocarcinoma	2.302	1.76E−12	8.555	Badea Pancreas [78]
ADAM19	Pancreatic Adenocarcinoma	3.348	1.79E−4	4.664	Lacobuzio-Donahue Pancreas 2 [36]
ADAMTS12	Pancreatic Adenocarcinoma	5.556	4.67E−20	12.338	Badea Pancreas [78]
	Pancreatic Adenocarcinoma	4.538	8.22E−4	5.149	Lacobuzio-Donahue Pancreas 2 [36]
ADAM12	Pancreatic Adenocarcinoma	1.848	0.002	3.564	Lacobuzio-Donahue Pancreas 2 [36]
	Pancreatic Adenocarcinoma	3.847	1.03E−9	6.811	Badea Pancreas [78]
ADAM10	Pancreatic Adenocarcinoma	2.302	1.67E−10	7.368	Badea Pancreas [78]
	Pancreatic Adenocarcinoma	1.968	0.004	4.008	Lacobuzio-Donahue Pancreas 2 [36]
ADAM15	Pancreatic Adenocarcinoma	2.591	4.06E−4	4.765	Lacobuzio-Donahue Pancreas 2 [36]

**Notes.**

PAAD Pancreatic Adenocarcinoma ADAM A Disintegrin and Metalloprotease Protein

### GO and pathway enrichment analysis

STRING (https://string-db.org/cgi/input.pl) is a web-based tool to provide an enriched analysis of known and predicted proteins (Szklarczyk et al., 2019). Now the STRING database (version 11.0) currently covers 24584628 proteins from 5090 organisms. We obtained the GO enrichment of ADAMs and their similar genes in three categories including Biological Process (BP), Molecular Function (MF) and Cellular Component (CC).

GSEALite (http://bioinfo.life.hust.edu.cn/web/GSCALite/) is a web-based platform with an integration of cancer genomics data of 33 cancer types from TCGA (Liu et al., 2018). We used the Pathway Activity module to present the difference of ADAMs expression between pathway activity groups.

### The protein–protein interaction of ADAMs and their similar genes in PAAD

Cytoscape is a bioinformatics software with multiple plugins to analyze and virtualize protein–protein interaction (PPI) and provide a molecular network connectivity (Shannon et al., 2003). We firstly obtained 20 similar genes of each ADAM gene in PAAD through an expression analysis in GEPIA 2.0 (http://gepia2.cancer-pku.cn/#index). Then we deleted duplicate gene and analyzed the PPI of ADAMs and their similar genes in STRING followed by a visualization processing in Cytoscape.

### Tumor grade correlation with ADAMs

UALCAN (http://ualcan.path.uab.edu) is an online web to perform in silico validation of potential genes of interest and valuable information, such as clinico-pathologic factors (Chandrashekar et al., 2017). Here we explored expression profile of ADAMs based on tumor grade. Box-Whisker plot showed a correlation between ADAMs and different tumor grade 1/2/3/4. The significance of difference was estimated by Student’s *t*-test considering unequal variance, and *P* < 0.05 was considered as statistically significant.

### Tumor immune estimation of ADAMs

TIMER (https://cistrome.shinyapps.io/timer/) is a comprehensive resource for systematical analysis of immune infiltrates with six immune infiltrates (B cells, CD4+ T cells, CD8+ T cells, Neutrophils, Macrophages, and Dendritic cells) (Li et al., 2016; Li et al., 2017). We used a correlation module of TIMER to draw the expression scatterplots between a pair of ADAMs with a purity-corrected partial Spearman’s rho value and statistical significance, as well as a pair of ADAMs with immune checkpoint blockade (ICB) immunotherapy-related molecules. These molecules have been previously reported as key targets of immune checkpoint inhibitors: programmed death 1 (PDCD1) and its ligand 1 (CD274, also known as PD-L1), and its ligand 2 (PDCD1LG2, also known as PD-L2), cytotoxic T-lymphocyte antigen 4 (CTLA4), T-cell immunoglobulin domain and mucin domain-containing molecule-3 (HAVCR2, also known as TIM-3), and indoleamine 2,3-dioxygenase 1 (IDO1). We then obtained a correlation of ADAMs expressions with immune infiltration levels. Subsequently, we used SCNA module to compare tumor infiltration levels with different somatic copy number alterations of ADAMs. The infiltration level for each SCNA category was compared with the normal using a two-sided Wilcoxon rank-sum test. We also used CNV module in GSEALite to analyze the copy number variations of ADAMs in PAAD.

UCSC Xena (http://xena.ucsc.edu) is an online exploration tool for integrating the any functional genomics data within the Xena Browser. We used Xena platform to search copy number of ADAMs in PAAD patient.

## Results

### Different expression of ADAMs family members in patients with PAAD

**Figure 1:**
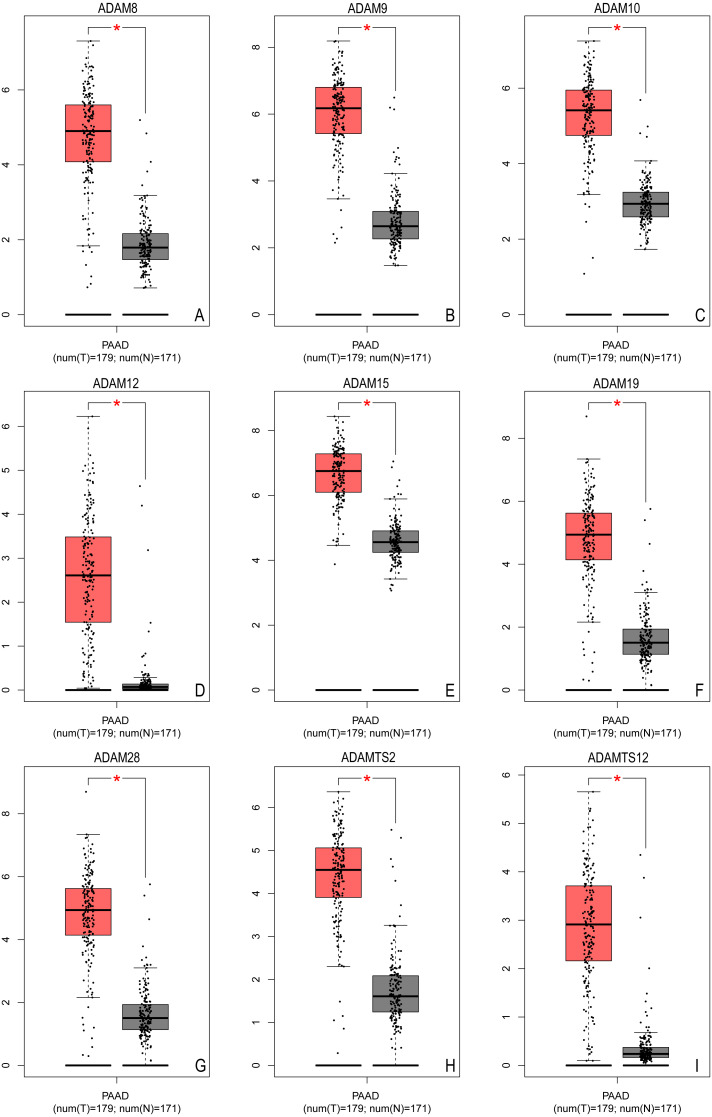
The expression of ADAMs genes in pancreatic adenocarcinoma (GEPIA based on TCGA database). The expression of distinct ADAMs genes was collected and compared between 179 tumor samples and 171 normal samples. (A) ADAM8, (B) ADAM9, (C) ADAM10, (D) ADAM12, (E) ADAM15, (F) ADAM19, (G) ADAM28, (H) ADAMTS2, (I) ADAMTS12. *p < 0.05.

The first section aimed to identify different ADAMs family members in PAAD. Based on TCGA database, we extracted more than 2600 DEGs in PAAD. Among these DEGs, significantly higher expression of ADAM8/9/10/12/15/19/28/TS2/TS12 were identified with log_2_FC from 2.194–3.53 (all *P* < 0.001) (Table 1), which implied that all these ADAMs were over-expressed in PAAD (Fig. 1).

We further verified the expression profile of these nine ADAMs in Oncomine database. There was more than one dataset from Oncomine to exhibit the over expression of all nine ADAMs in PAAD (Table 2). These data established that nine ADAMs genes were aberrantly activated in patients with PAAD.

### Correlation analysis of ADAMs in PAAD patient

After the expression of 9 ADAMs was found to be an elevated level in PAAD patients, we next investigated the correlation of each other between ADAMs. It was apparently showed that most of the nine ADAMs expression were significantly correlated to each other. However, ADAM 15 expression was no significant correlation with ADAM10/12/19/TS2TS12, the same as ADAM28 expression with ADAM12/19/TS2 and ADAM8 expression with ADAMTS2, separately (Fig. S1). Based on the expression and correlation analysis, our results provided evidence to regard ADAM8/9/10/12/15/19/28/TS2/TS12 as one cluster of over expression DEGs during the progression of PAAD. The next section, therefore, moved on to discuss the biological functions and pathways of the nine ADAMs family members.

### Pathways enrichment of ADAMs in PAAD

**Figure 2:**
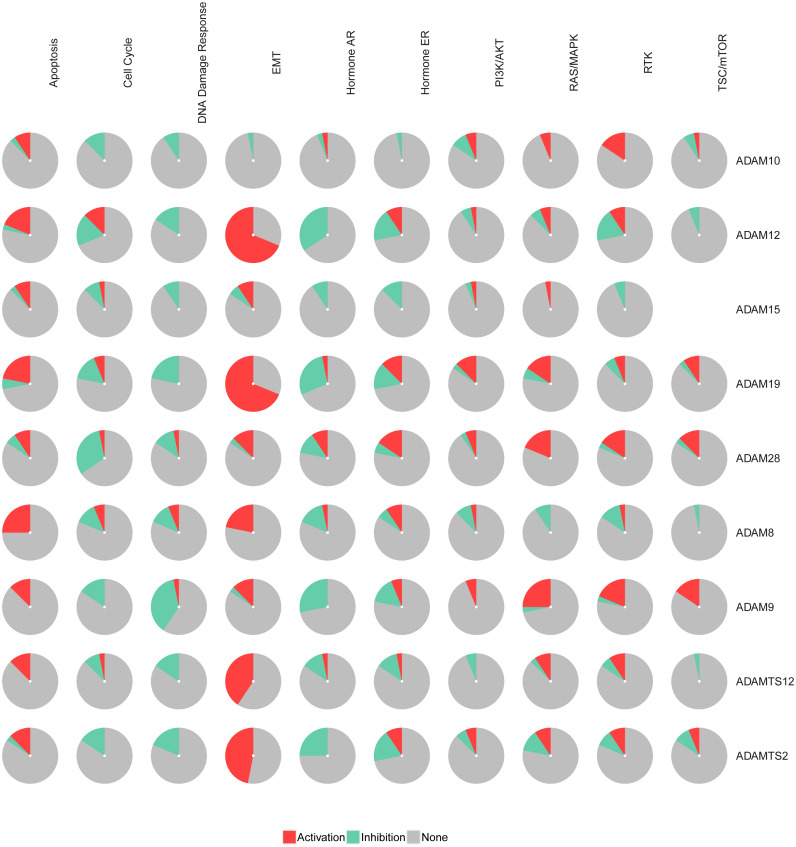
The pathway enrichment analysis of nine ADAMs genes (GSEALite). Each ADAMs member was enriched in 10 pathways. Red in the pie chart was the proportion of activation. Green in the pie chart was the proportion of inhibition.

The pathways of the nine ADAMs were enriched with GSCALite. We found that 10 pathways were associated with the function of the nine ADAMs in PAAD. The expressions of ADAM8/12/19/TS2/TS12 were in a remarkably high level in epithelial-mesenchymal transition (EMT) among 10 pathways (Fig. 2). The expressions of nine ADAMs were almost inhibited in DNA damage repair, cell cycle, and hormone androgen receptor (AR) related pathway. Taken together, these results showed the biological function and pathways of the nine ADAMs in PAAD, further suggesting ADAMs were implicated in extracellular physiological processes.

### PPI and GO analysis of ADAMs and their similar genes in PAAD

**Figure 3:**
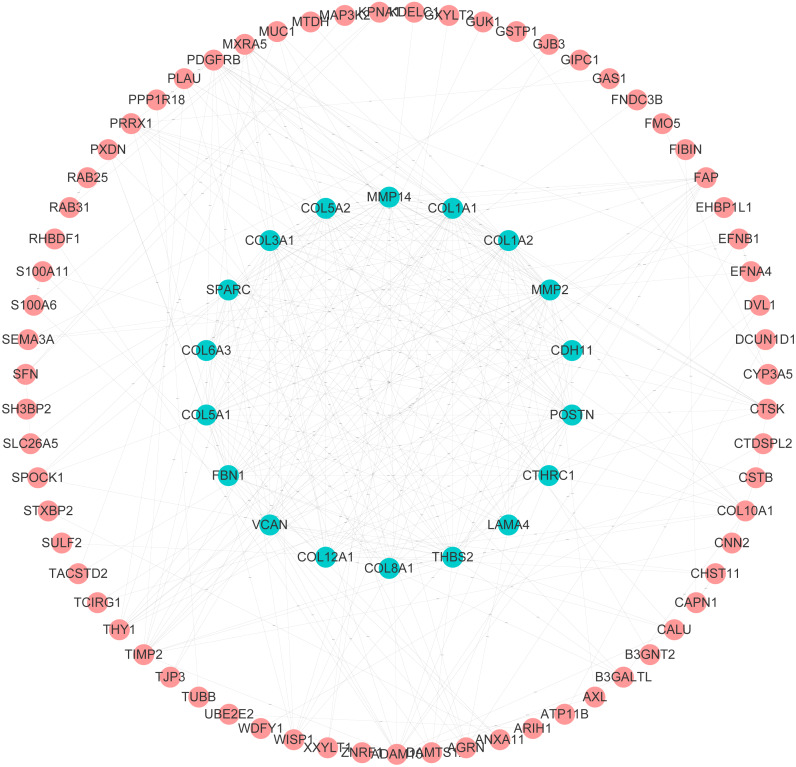
Protein–protein interaction analysis of ADAMs and their similar genes in PAAD (GEPIA 2.0). The level of PPI connectivity in the inner circle was higher than that in the outer circle.

After analyzing biological functions of ADAMs, we continued to investigate the similar genes of ADAMs in PAAD, which play synergistic roles in PAAD development. The network analysis of PPI illustrated that the similar genes of ADAMs had a dramatical connectivity with each other, which validated that there was a synergistic function of similar genes (Fig. 3). GO analysis revealed that similar genes of ADAMs were remarkably related to the extracellular matrix organization and cell adhesion (Table S1). And these similar genes were significantly associated with cellular component, including extracellular matrix, extracellular region part, extracellular matrix component, collagen-containin, extracellular matrix, and extracellular region (Table S1). Our results provided more evidence for the role of ADAMs and their similar genes in extracellular biological function.

### Correlation between ADAMs expression and tumor grade in PAAD

**Figure 4:**
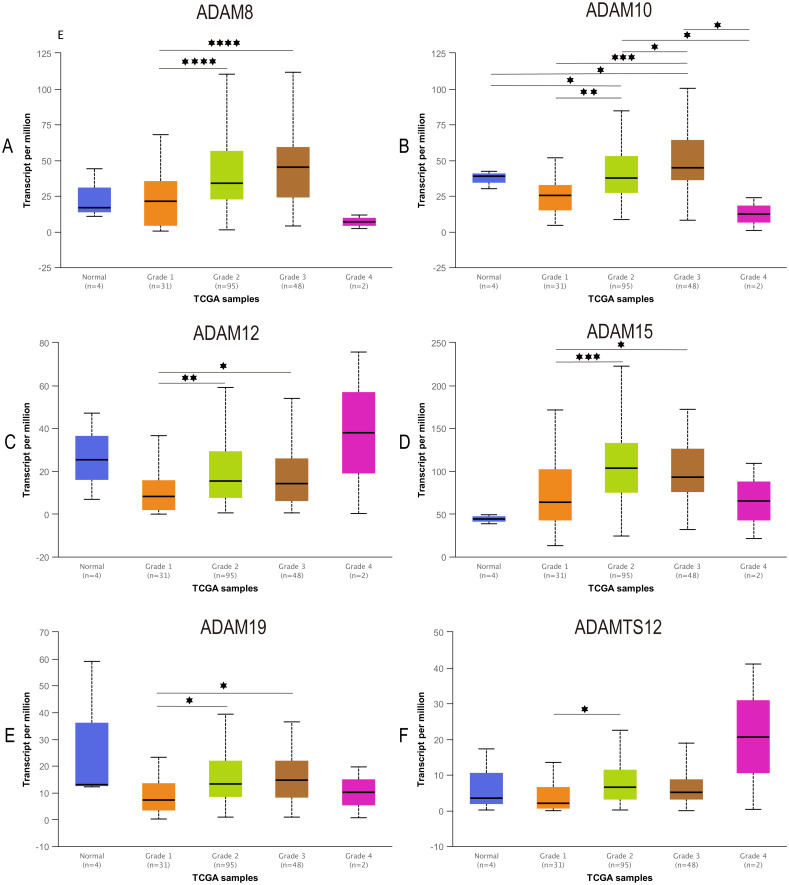
Association of expression of distinct ADAMs family members with tumor grades of PAAD patients (UALCAN). The expression of ADAMs was compared between normal and grades of PAAD. (A) ADAM8, (B) ADAM10, (C) ADAM12, (D) ADAM15, (E) ADAM19, (F) ADAMTS12. *p < 0.05, **p < 0.01, ***p < 0.001, and ****p < 0.0001.

To further our understanding of the role of ADAMs in the development of PAAD, we assessed the expression of ADAMs based on tumor grade for PAAD. We found that varying grades of tumor were significantly correlated with 6 ADAMs, including ADAM8/10/12/15/19/TS12 (Fig. 4). The expression of ADAM8/12/15/19 was apparently increased in the comparison between grade 1 and grade 2/3. Interestingly, ADAM10 presented almost a grade-wide influence with statistical significance. In short, we concluded that some ADAMs played a potential role on tumorigenesis and development of PAAD.

### Correlation between ADAMs expressions and immune infiltration levels in PAAD

**Figure 5:**
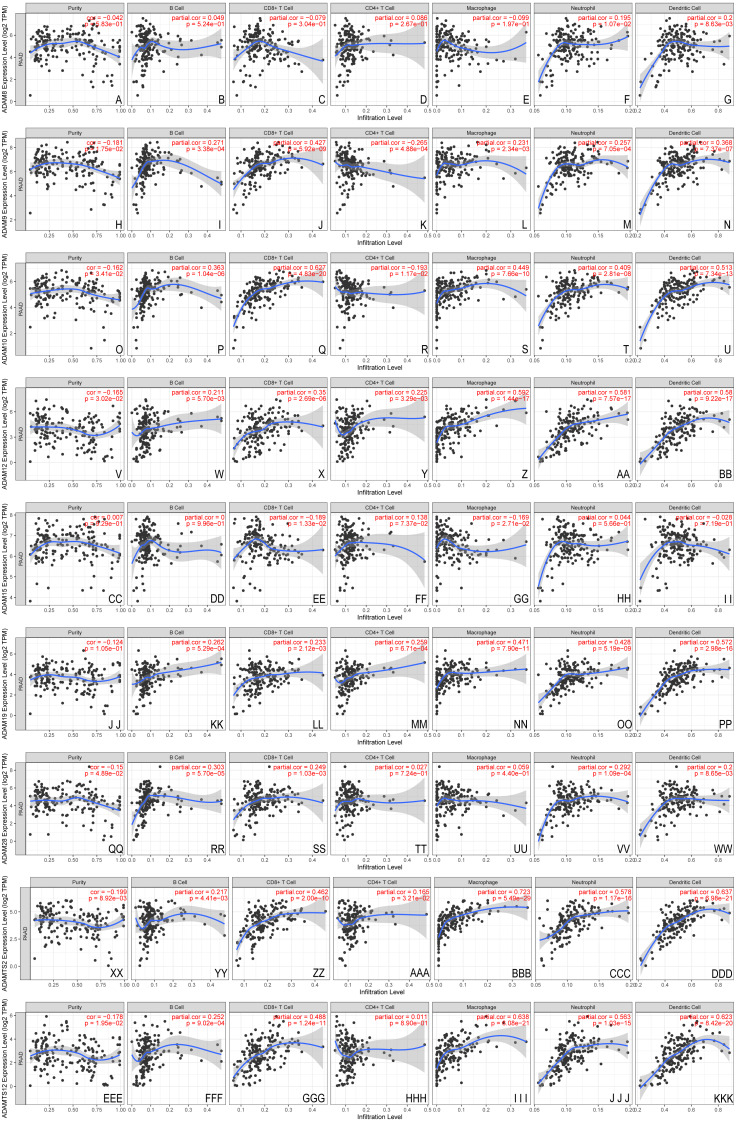
The immune infiltrates estimation of distinct ADAMs genes (TIMER). The correlation of 6 immunocytes with ADAMs was estimate. (A–I) ADAM8, (H–N) ADAM9, (O–U) ADAM10, (V–BB) ADAM12, (CC–II) ADAM15, (JJ–PP) ADAM19, (QQ–WW) ADAM28, (XX–DDD) ADAMTS2, (EEE–KKK) ADAMTS12. Cor > 0 means a positive correlation, Cor < 0 means a negative correlation. *p* < 0.01 represents a significant correlation.

Given the potential role of ADAMs in the cell-environment interaction, we performed a correlation of ADAMs expression with immune infiltration level in PAAD (Fig. 5). When there was no immune infiltrate in PAAD, 8 ADAMs had a different expression with no significance, except ADAMTS12 that was negatively related to purity state of tumor cell. The expression of 8 ADAMs positively correlated with B cell without ADAM15. Intriguingly, ADAM15 level had no significant variation with either of 6 immune infiltrates. Except for ADAM15, the other eight ADAMs had remarkably positive correlations with almost five immune infiltrates, especially dendritic cell. ADAM8 expressed differently with only dendritic cell. These results provided important insights into the correlation between ADAMs and immune infiltration in PAAD patients.

**Figure 6:**
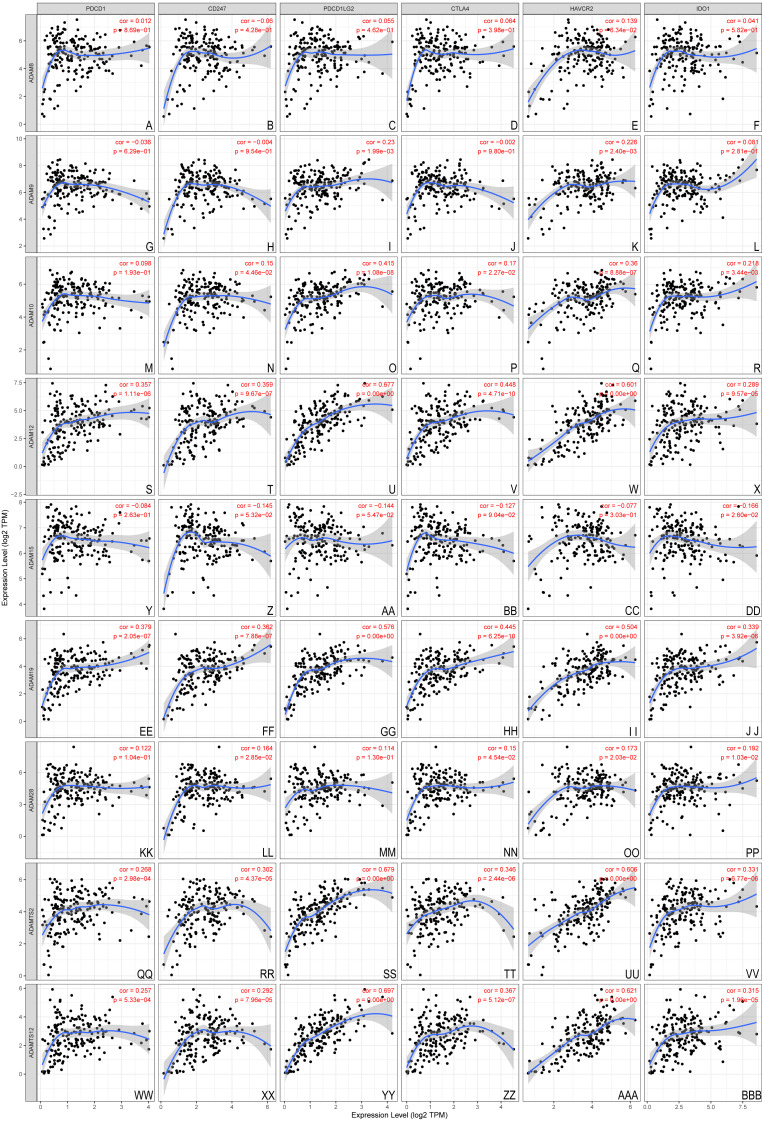
Association between ADAMs and crucial immune checkpoint genes (TIMER). The correlation of 6 immunotherapy-related molecules was estimated with ADAMs. (A–E) ADAM8, (G–L) ADAM9, (M–R) ADAM10, (S–X) ADAM12, (Y–DD) ADAM15, (EE–JJ) ADAM19, (KK–PP) ADAM28, (QQ–VV) ADAMTS2, (WW–BBB) ADAMTS12. Cor > 0 means a positive correlation, Cor < 0 means a negative correlation. *p* < 0.001 represents a significant correlation.

The association between nine ADAMs and ICB therapy-related genes was performed to assess the possible role of ADAMs family members in the immunotherapy of ICB in PAAD (Fig. 6). ADAM 12/19/TS2/TS12 were positively related to all six ICB immunotherapy-related molecules (cor > 0, *P* < 0.001), whereas ADAM8/9/15/28 had no relevance with either of six molecules. There was a forward trend of all six molecules with ADAMs, except ADAM15. Our findings implied a possible role of ADAMs in ICB therapy.

### Immune infiltration level in PAAD with copy number variations of ADAMs

**Figure 7:**
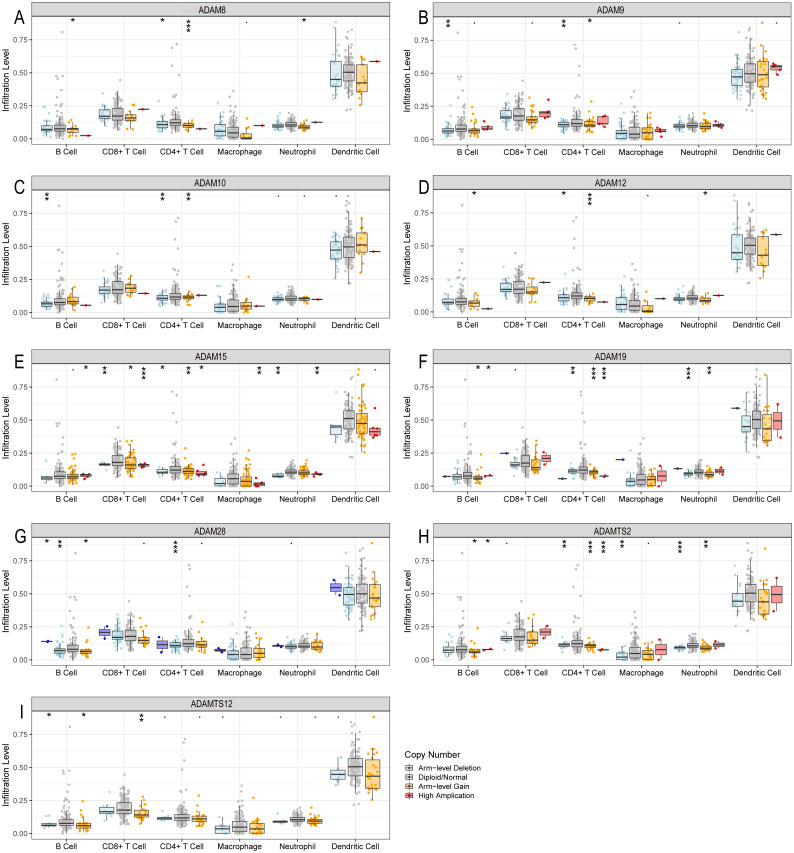
The comparison of tumor infiltration levels in PAAD with different somatic copy number alterations for ADAMs (TIMER). (A) ADAM8, (B) ADAM9, (C) ADAM10, (D) ADAM12, (E) ADAM15, (F) ADAM19, (G) ADAM28, (H) ADAMTS2, (I) ADAMTS12. *p < 0.05, **p < 0.01, and ***p < 0.001.

Regarding that different expressions of ADAMs involved in the regulation of immune infiltration, we investigated the somatic copy number variation of ADAMs in PAAD (Fig. 7). We found that an arm-level alteration of ADAM8/9/10/12/19/28/TS2 genes attenuated the immune infiltration of B cell and CD4+T cell. An arm-level alteration of ADAM8/12/19/TS2 genes also decreased the immune infiltration of neutrophil. The high amplification of ADAM15 gene influenced the infiltration levels of B cells, CD4+ T cells, CD8+ T cells, Neutrophils, and Macrophages. And an arm-level alteration of ADAM15 gene decreased the immune infiltration levels of CD4+ T cells, CD8+ T cells, and Neutrophil. Interestingly, dendritic cells had no influence by CNV of ADAMs genes. These results suggested that B cell and CD4+T cell infiltrates were particularly affected by CNV of ADAMs in patient with PAAD.

**Figure 8:**
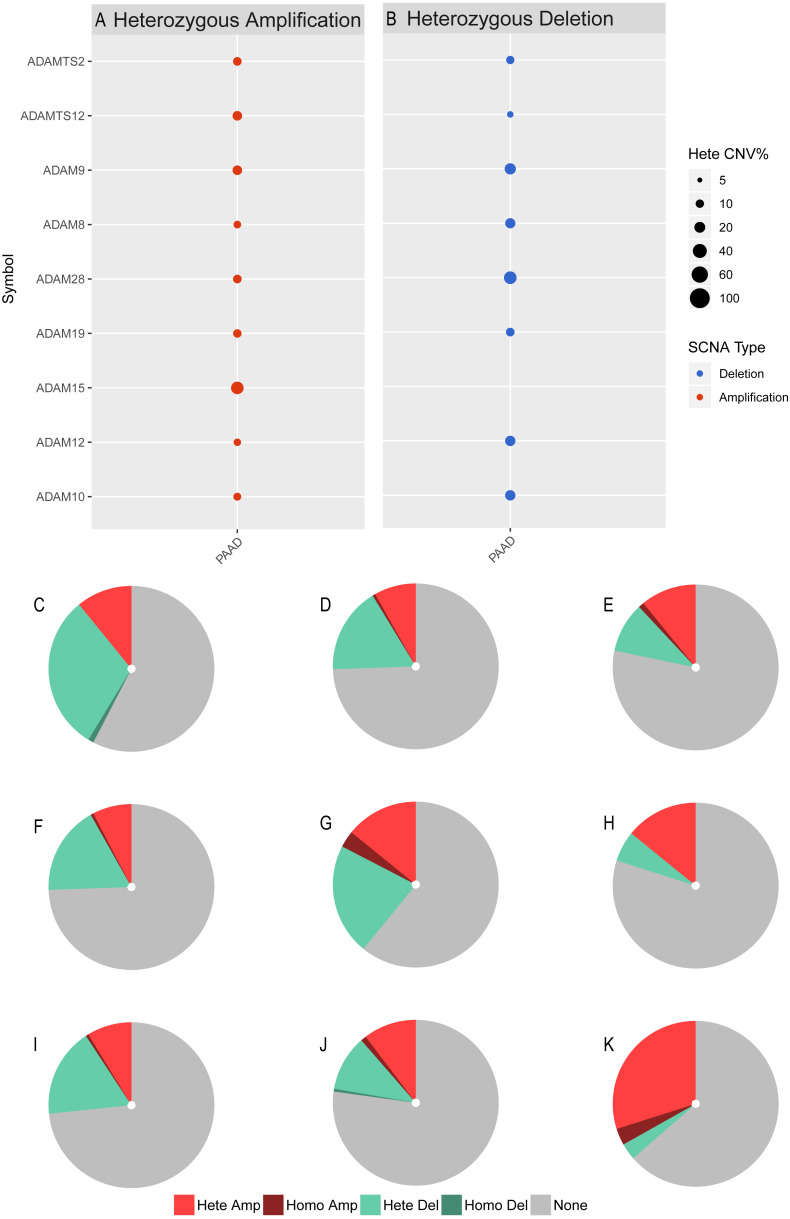
CNV analysis of distinct ADAMs genes in PAAD (GSEALite). (A) Heterozygous Amplification, (B) Deletion for distinct ADAMs genes. (C) ADAM28, (D) ADAM8, (E) ADAMTS2, (F) ADAM12, (G) ADAM9, (H) ADAMTS12, (I) ADAM10, (J) ADAM19, (K) ADAM15.

**Figure 9:**
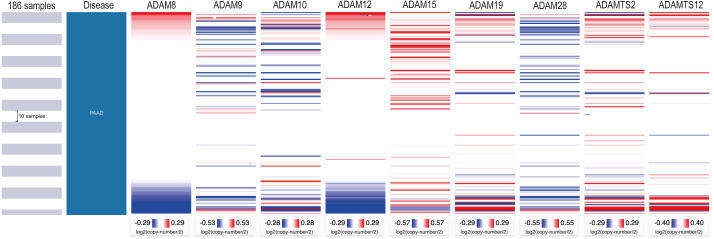
Copy number analysis of distinct ADAMs genes in PAAD (UCSC Xena).

We next investigated the CNV of ADAMs in PAAD. An analysis of CNV% showed that a heterozygous variation of ADAM15 gene accounted for a most heterozygous amplification without deletion. ADAM9/28 genes presented a relatively high probability of heterozygous deletion (Fig. 8). We also used Xena to analyze copy numbers of ADAMs from 186 samples from TCGA (Fig. 9). In some PAAD samples, the copy number of ADAM9 increased more than 2.8 times, as well as those of ADAM 15/28. ADAM15 had a most increase of copy number and ADAM28 had a most decrease in PAAD samples, which were directly in line with the results from the CNV% analysis (Fig. 8). As a result, CNV of ADAMs family members held their own variations, which may influence immune infiltration level in PAAD.

## Discussion

The identification of ADAMs in a collection of data derived from TCGA and Oncomine databases confirms the involvement of these genes in development and progression of PAAD. Despite some ADAMs have been reported to play important roles in tumor, distinct role of ADAMs family members in PAAD awaits clarification. In this study, the expression of ADAMs was identified followed by an analysis of their biological functions. Given that ADAMs had a potential influence on the cell-environment interaction, the correlation between ADAMs and immune infiltration in PAAD was established and analyzed. The CNV in ADAMs genes influenced distinct immune infiltrates including different types of immunocytes.

Previous studies revealed the biological function of ADAMs, which raised the possibility that these nine ADAMs interacted with the extracellular environment during the progression of PAAD. In this study, ADAMs were found to be apparently involved in the regulation of tumor immune infiltration. Among 9 ADAMs, only ADAM15 correlated with neither tumor purity nor any immune infiltrate. ADAM15 was reported to play a contradictory role in tumor metastasis (Kobayashi & Watabe, 2008; Najy, Day & Day, 2008; Ungerer et al., 2010). On the one hand, ADAM15 promoted cell adhesion so as to decrease tumor cell migration. On the other hand, ADAM5 was found to facilitate the invasion and migration of tumor. The concrete function of ADAM15 is still to be determined. Except for ADAM15, ADAM8/9/10/12/19/28/TS2/TS12 were all significantly positive related to dendritic cell. DC is professional antigen presenting cell (APC) that can capture tumor-associated antigens and provide antigens and co-stimulatory signals to immunocytes in adaptive immune system (Hansen & Andersen, 2017; Veglia & Gabrilovich, 2017). In the current study, overexpression of 8 ADAMs significantly improved an infiltration of DC, which elucidated the important role of ADAMs at the initial stage of tumor immunity. In addition to DC, 4 types of immunocytes including B Cell, neutrophil, CD8+T Cell, and Macrophage were positively affected by the upregulation of distinct ADAMs.

The results from pathway enrichment presented an obvious relationship of ADAMs with EMT. The majority of ADAMs family members involved in EMT-related pathway. The transition from epithelial to mesenchymal statue is a characteristic of malignancy, promoting tumor invasion (Li et al., 2020). Additionally, the occurrence of EMT can inhibit the activation of immune cells and promote the proliferation of regulatory immune cells, which finalizes the tumor immunosuppression. It has been reported that the invasion ability of cancer cell increased after tumor-associated macrophages (TAMs)-conditioned medium (Guo et al., 2015). CD247, an inhibitory molecule expressed by antigen-presenting cells, can enable T cells to be inactivated and lose immune function after binding with the corresponding receptor PDCD1 on the surface of T cells. the high expressions of PDCD1 and CD247 were generally associated with the change in expression of EMT biomarkers (Alsuliman et al., 2015; Kim et al., 2016). Given that majority of ADAMs involved in EMT pathway, we implied that ADAMs family played a promotive role in the process of immune infiltration.

Here we found that B cell was positively related to all 8 ADAMs, except ADAM8 with no significance. A recent research found that the biomarker of B cell was expressed differently between melanoma patients with and without a sensitivity of the immune checkpoint therapy (Helmink et al., 2020). Another studies from French and Sweden also demonstrated a positive role of B cell in immunotherapy response, especially with the evaluation of tertiary lymphoid structure (TLS), which were aggregates of immune cells formed near the sites of tumorous tissues (Cabrita et al., 2020; Petitprez et al., 2020). Our study provided a clue that ADAMs, which held an elevated expression during B cell infiltration, released various signals from cell surface to improve communication between tumor cell and B cell for the regulation of immunotherapy response.

Similar to B cell in this study, neutrophils and CD8+T cell held an extensive correlation with over expression of ADAMs family members, including ADAM9/10/12/19/28/TS2/TS12. Neutrophil, which accounted for a largest proportion of white cells in peripheral blood, could kill tumor cells through secreting cytokines and reactive oxygen species (Sionov et al., 2019). There were differing reports reflecting both anti- and pro-tumor role of neutrophil. Therefore, further studies are still required to assess the exact role of ADAMs on tumor infiltrating neutrophils in the tumor microenvironment. CD8+T cell played a direct and clear role on tumor cell (Giese, Hind & Huttenlocher, 2019; Okada et al., 2020; Taya et al., 2020). Previous studies showed that CD8+T cell infiltration improved a survival benefit of the patient with advanced cancer (Vihervuori et al., 2019). While activated, CD8+T cell induced tumor cell to express some factor to recruit macrophage, which weakened the efficacy of PDCD1 immunotherapy (Neubert et al., 2018). That evidence might explain our results that macrophage infiltration was also significantly relevant to a high expression of some ADAMs family members.

With an application of ICB inhibitor, tumor immunotherapy has become a promising approach for cancer treatment (Champiat et al., 2016; Goodman, Patel & Kurzrock, 2017). Thus, it was essential to explore the mechanism underlying the regulation of ICB inhibitor (Mushtaq et al., 2018). The molecule we collected were the key targets of immune checkpoint inhibitors (Zhang et al., 2020). They were all involved in the immune checkpoint interaction (Ribas & Wolchok, 2018). Here we constructed a correlation of ADAMs with key targets of ICB inhibitors. ADAM 12/19/TS2/TS12 was found to be positively related with all 6 crucial targets of ICB inhibitors, suggesting that a cluster of 4 ADAMs might played a potential role in response to ICB immunotherapy for patient with PAAD.

Focusing on the CNV of ADAMs in patient with PAAD may help us to interpret an impact of ADAMs expression on immune infiltration. CNV, a segment of DNA with copy number differences in the comparison of two or more genomes, resulted in different levels of gene expression. In our study, arm-level gain was the most part of CNV. The infiltration level of B cell was declined by the arm-level gain of CD8+T ADAM8/12/19/28/TS2/TS12 genes, as well as CD4+T cell infiltration by the arm-level gain of ADAM8/9/10/12/15/19/TS12 and neutrophil infiltration by the arm-level gain of ADAM8/12/19/TS2. Arm-level deletion of ADAMs genes was also an important CNV for the regulation of immunocytes, including B cell, CD4+T cell, CD8+T cell, macrophage, and neutrophil. One unanticipated finding was that DC infiltration was not interfered by either of CNVs in ADAMs genes. Given our results of DC infiltration influenced by 8 ADAMs, there may be other putative regulations underlying interaction between DC infiltration and ADAMs.

According to an immunocytes estimation, ADAMs were potentially implicated in the process of immune infiltration. In the decades, the immunotherapy was applied based on the mechanism research, such as immune infiltration, immune evasion and immune surveillance. These immune activities actually impact the quality of cancer therapy. In 2019, Zhang and his team found that CD38/CD101 co-repression on tumor-infiltrating lymphocytes were related with a poor survival in PDAC patients (Zhang et al., 2019). Zhao et al. reported that patients with high level of tumor-infiltrating CD8+T cells had a longer overall survival time (OS) than those with low level of tumor-infiltrating CD8+T cells. Tumor infiltration FOXP3+ cells correlated with reduced survival time and reduced relapse free survival (RFS). The sample group with a high level of myeloid-derived suppressor cell showed a significantly shorter OS and RFS than the group with a low level of myeloid-derived suppressor cell (Zhao et al., 2018). The in vivo experiment in C57BL/6 mouse model showed that Treg and a depletion of myeloid-derived suppressor cells significantly decreased the volume of pancreatic cancer (Zhao et al., 2018). The observation that ADAMs family members exhibited a different positive association with immune infiltration enabled them as biomarker of immune infiltrates or even therapeutic targets. With our results, these recent reports revealed that immune infiltration played a potential role on the survival probability of PAAD, suggesting the immunotherapy with ADAMs might be available for the clinical administration of pancreatic cancer in future.

In this study, we attempted to evaluate the prognostic value of ADAMs family members in PAAD. One interesting finding was that the expression of the nine ADAMs was remarkably lower in Grade 1 than that of ADAMs in Grade 2/3 but not in Grade 4. This result partially reflected that ADAMs expressions were enhanced along with the progression of PAAD. When PAAD gradually turned into the most malignant grade, tumor cell preferred to update its response to extracellular interaction through some putative mechanism underlying a regulation of signal exposure, such as ADAMs. There is another explanation that insufficient data from PAAD patient caused no significant comparison between Grade 4 and other Grades. As a result, further study is warranted so that we can figure out the role of ADAMs in the development of PAAD.

Although our study was based on the data that was derived from TCGA database and verified by Oncomine database, the impact of ADAMs family members on immune infiltration should be further measured in solid experiments to provide a practical evidence. Another limitation of this study was still lack of patient sample. Results from tumor grade analysis showed that we did not obtain a significant difference between grade 4 and other grades from very few samples. In addition, we examined transcriptional expressions of 9 ADAMs from DEGs in PAAD, hence the protein expressions of ADAMs family members should be determined in the future.

## Conclusion

**Figure 10:**
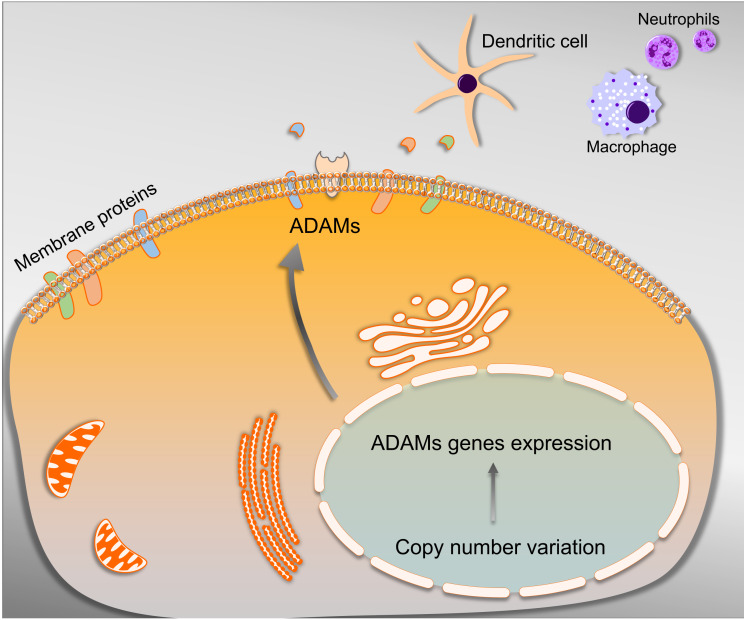
Schematic representation of the mechanism underlying that ADAMs family members were able to cleave cell membrane proteins under the mediation of CNV, which contributed to releasing substrates for advancing the tumor immune infiltration.

The current study suggested that overexpression of nine ADAMs family members should be labeled as a promising cluster of biomarkers when the PAAD patient undertook immunotherapy. The wide spectrum of ADAMs’ substrates on the one hand is matched by the complexity of cell-environment interaction that enabled the regulation of immune infiltration on the other. The ADAMs harboring a significant correlation with ICB therapy-related molecules were potential to become a cluster of targets in ICB therapy for PAAD. Furthermore, CNV of ADAMs genes were found to be a significantly associated with the immune infiltrate alteration, which provided a better understanding of molecular targets for improved immunotherapy strategies in the future (Fig. 10). Therefore, the above results may provide guidance for developing novel therapy combined with immunotherapy for PAAD.

##  Supplemental Information

10.7717/peerj.9736/supp-1 Supplemental Information 1 Correlation between the expression of 9 ADAMs genes (TIMER)

10.7717/peerj.9736/supp-2 Supplemental Information 2 GO analysis for similar genes of 9 ADAMs family members

## Additional Information and Declarations

### Data Availability

The following information was supplied regarding data availability: Data are available at TCGA https://portal.gdc.cancer.gov/projects/TCGA-PAAD (search terms: TCGA-CESC and TCGA-PAAD) and Oncomine (target genes isolated and collected using TCGA and Oncomine are available in Tables 1 and 2, separately).
