# Supplementary figures and images for: The nine ADAMs family members serve as potential biomarkers for immune infiltration in pancreatic adenocarcinoma
Source: PeerJ. 2020 Sep 30;8:e9736. doi: 10.7717/peerj.9736 (PMC7532768; doi:10.7717/peerj.9736)

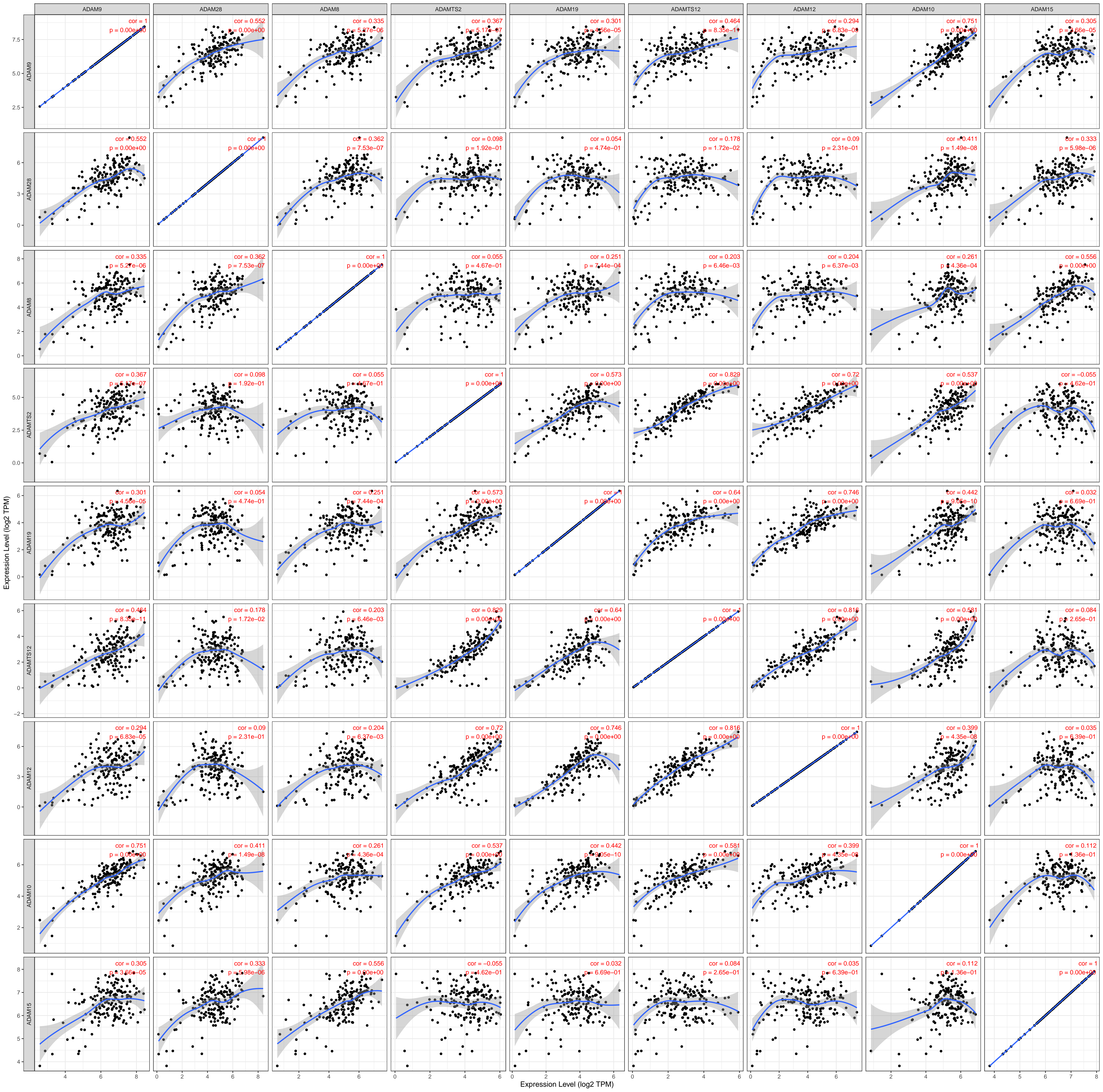

Supplement: Supplemental Information 1 [file peerj-08-9736-s001.pdf]
