# Supplementary material for: The nine ADAMs family members serve as potential biomarkers for immune infiltration in pancreatic adenocarcinoma
Source: PeerJ. 2020 Sep 30;8:e9736. doi: 10.7717/peerj.9736 (PMC7532768; doi:10.7717/peerj.9736)
Supplement: Supplemental Information 2 [file peerj-08-9736-s002.docx]

Table supplement 1. GO analysis for similar genes of 9 ADAMs family members

| **Categories** | **Term ID** | **Term description** | **False discovery rate** | **Matching proteins in your network (labels)** |
| --- | --- | --- | --- | --- |
| **Cellular Component** | GO:0031012 | extracellular matrix | 1.81E-10 | ENSP00000225964,ENSP00000230538,ENSP00000231061,ENSP00000252804,ENSP00000261037,ENSP00000295550,ENSP00000297268,ENSP00000304408,ENSP00000308208,ENSP00000325146,ENSP00000325527,ENSP00000327368,ENSP00000330523,ENSP00000355751,ENSP00000360882,ENSP00000364000,ENSP00000368678,ENSP00000369071,ENSP00000422554 |
|  | GO:0044421 | extracellular region part | 1.70E-09 | ENSP00000188790,ENSP00000219070,ENSP00000225964,ENSP00000230538,ENSP00000231061,ENSP00000250160,ENSP00000252804,ENSP00000261037,ENSP00000262768,ENSP00000264094,ENSP00000265362,ENSP00000271651,ENSP00000291568,ENSP00000295550,ENSP00000297268,ENSP00000301178,ENSP00000304408,ENSP00000308208,ENSP00000325146,ENSP00000325527,ENSP00000327368,ENSP00000330523,ENSP00000340989,ENSP00000353007,ENSP00000355751,ENSP00000360269,ENSP00000360882,ENSP00000361850,ENSP00000362095,ENSP00000364000,ENSP00000368678,ENSP00000369071,ENSP00000378401,ENSP00000403902,ENSP00000422554,ENSP00000484824 |
|  | GO:0044420 | extracellular matrix component | 6.97E-09 | ENSP00000225964,ENSP00000230538,ENSP00000261037,ENSP00000297268,ENSP00000304408,ENSP00000325146,ENSP00000325527,ENSP00000360882,ENSP00000364000,ENSP00000368678 |
|  | GO:0062023 | collagen-containing extracellular matrix | 8.79E-09 | ENSP00000225964,ENSP00000230538,ENSP00000231061,ENSP00000261037,ENSP00000297268,ENSP00000304408,ENSP00000325146,ENSP00000325527,ENSP00000355751,ENSP00000360882,ENSP00000364000,ENSP00000368678,ENSP00000422554 |
|  | GO:0005576 | extracellular region | 1.84E-08 | ENSP00000161006,ENSP00000188790,ENSP00000219070,ENSP00000225964,ENSP00000230538,ENSP00000231061,ENSP00000250160,ENSP00000252804,ENSP00000261037,ENSP00000262768,ENSP00000263097,ENSP00000264094,ENSP00000265362,ENSP00000271651,ENSP00000284240,ENSP00000291568,ENSP00000295550,ENSP00000297268,ENSP00000301178,ENSP00000304408,ENSP00000308208,ENSP00000321962,ENSP00000325146,ENSP00000325527,ENSP00000327368,ENSP00000330523,ENSP00000330601,ENSP00000339001,ENSP00000340989,ENSP00000353007,ENSP00000355751,ENSP00000360269,ENSP00000360882,ENSP00000361850,ENSP00000362095,ENSP00000364000,ENSP00000368678,ENSP00000369071,ENSP00000378401,ENSP00000381607,ENSP00000403902,ENSP00000413606,ENSP00000414378,ENSP00000422554,ENSP00000431984,ENSP00000438248,ENSP00000484824 |
| **Molecular Function** | GO:0048407 | platelet-derived growth factor binding | 3.21E-05 | COL1A1,COL1A2,COL3A1,COL5A1,PDGFRB |
|  | GO:0005178 | integrin binding | 9.39E-05 | ADAM10,COL3A1,COL5A1,FAP,FBN1,MMP14,THY1,TIMP2,WISP1 |
|  | GO:0019838 | growth factor binding | 9.39E-05 | COL1A1,COL1A2,COL3A1,COL5A1,PDGFRB,PXDN,RHBDF1,SRPX2,WISP1 |
|  | GO:0050839 | cell adhesion molecule binding | 0.00031 | ADAM10,COL3A1,COL5A1,FAP,FBN1,MMP14,PVRL4,THY1,TIMP2,WISP1 |
|  | GO:0005201 | extracellular matrix structural constituent | 0.0019 | COL1A2,COL3A1,FBN1,LAMA4,PXDN,VCAN |
| **Biological Process** | GO:0030198 | extracellular matrix organization | 2.02E-13 | ADAM10,AGRN,COL10A1,COL12A1,COL1A1,COL1A2,COL3A1,COL5A1,COL5A2,COL6A3,COL8A1,CTSK,FBN1,LAMA4,LOXL3,MMP14,MMP2,POSTN,PXDN,SPARC,SULF2,TIMP2,VCAN |
|  | GO:0007155 | cell adhesion | 4.39E-05 | ADAMTS12,ANTXR1,AXL,CDH11,CERCAM,COL12A1,COL3A1,COL5A1,COL6A3,COL8A1,EFNB1,FAP,FBN1,LAMA4,POSTN,PVRL4,SPOCK1,SRPX2,TACSTD2,THBS2,THY1,VCAN,WISP1 |
|  | GO:0016043 | cellular component organization | 7.36E-05 | ADAM10,AGRN,ANTXR1,ATP11B,AXL,B3GNT2,BICD2,CDC73,CDH11,CNIH1,CNN2,COL10A1,COL12A1,COL1A1,COL1A2,COL3A1,COL5A1,COL5A2,COL6A3,COL8A1,CORO1B,CTHRC1,CTSK,DVL1,EFNA4,EFNB1,EIF2S1,FBN1,GPR35,INF2,KCNN4,KCTD9,KPNA1,LAMA4,LIMS1,LOXL3,MAP7D1,MMP14,MMP2,MTDH,NAA50,PDGFRB,POSTN,PVRL4,PXDN,RAB25,RAB31,RSF1,S100A6,SEMA3A,SFN,SLAIN2,SLC26A5,SLX1A,SNX6,SPARC,SPTY2D1,SULF2,TCIRG1,THY1,TIMP2,TMOD3,TSPO,TUBB,VCAN,ZNF148 |
|  | GO:0009653 | anatomical structure morphogenesis | 7.65E-05 | ANTXR1,AXL,B3GNT2,CAPN1,CDC73,CDH11,CHST11,COL12A1,COL1A1,COL1A2,COL3A1,COL5A1,COL5A2,COL8A1,CTHRC1,DVL1,EFNA4,EFNB1,ELK3,FAP,FBN1,KIF26B,MMP14,MMP2,PDGFRB,POSTN,PPP1R13L,PRRX1,RAB25,S100A6,SEMA3A,SRPX2,TACSTD2,THY1,TMOD3,ZFPM2 |
|  | GO:0071840 | cellular component organization or biogenesis | 8.26E-05 | ADAM10,AGRN,ANTXR1,ATP11B,AXL,B3GNT2,BICD2,CDC73,CDH11,CNIH1,CNN2,COL10A1,COL12A1,COL1A1,COL1A2,COL3A1,COL5A1,COL5A2,COL6A3,COL8A1,CORO1B,CTHRC1,CTSK,DVL1,EFNA4,EFNB1,EIF2S1,FBN1,FCF1,GPR35,INF2,KCNN4,KCTD9,KPNA1,LAMA4,LIMS1,LOXL3,MAP7D1,MMP14,MMP2,MTDH,NAA50,PDGFRB,POSTN,PVRL4,PXDN,RAB25,RAB31,RSF1,S100A6,SEMA3A,SFN,SLAIN2,SLC26A5,SLX1A,SNX6,SPARC,SPTY2D1,SULF2,TCIRG1,THY1,TIMP2,TMOD3,TSPO,TUBB,VCAN,ZNF148 |

ADAM: A Disintegrin and Metalloprotease Protein.
